# JNK3 Maintains Expression of the Insulin Receptor Substrate 2 (IRS2) in Insulin-Secreting Cells: Functional Consequences for Insulin Signaling

**DOI:** 10.1371/journal.pone.0035997

**Published:** 2012-05-01

**Authors:** Saida Abdelli, Christophe Bonny

**Affiliations:** Service of Medical Genetics, Centre Hospitalier Universitaire Vaudois (CHUV), Lausanne, Switzerland; Universita Magna-Graecia di Catanzaro, Italy

## Abstract

We have recently shown that silencing of the brain/islet specific c-Jun N-terminal Kinase3 (JNK3) isoform enhances both basal and cytokine-induced beta-cell apoptosis, whereas silencing of JNK1 or JNK2 has opposite effects. While it is known that JNK1 or JNK2 may promote apoptosis by inhibiting the activity of the pro-survival Akt pathway, the effect of JNK3 on Akt has not been documented. This study aims to determine the involvement of individual JNKs and specifically JNK3 in the regulation of the Akt signaling pathway in insulin-secreting cells. JNK3 silencing strongly decreases Insulin Receptor Substrate 2 (IRS2) protein expression, and blocks Akt2 but not Akt1 activation by insulin, while the silencing of JNK1 or JNK2 activates both Akt1 and Akt2. Concomitantly, the silencing of JNK1 or JNK2, but not of JNK3, potently phosphorylates the glycogen synthase kinase3 (GSK3β). JNK3 silencing also decreases the activity of the transcription factor Forkhead BoxO3A (FoxO3A) that is known to control IRS2 expression, in addition to increasing c-Jun levels that are known to inhibit insulin gene expression. In conclusion, we propose that JNK1/2 on one hand and JNK3 on the other hand, have opposite effects on insulin-signaling in insulin-secreting cells; JNK3 protects beta-cells from apoptosis and dysfunction mainly through maintenance of a normal IRS2 to Akt2 signaling pathway. It seems that JNK3 mediates its effects mainly at the transcriptional level, while JNK1 or JNK2 appear to mediate their pro-apoptotic effect in the cytoplasm.

## Introduction

Sustained pancreatic beta-cell death, which mainly occurs by apoptosis, ultimately leads to diabetes mellitus [1]–[3]. Apoptosis follows an autoimmune process called insulitis that involves secretion of a number of pro-inflammatory cytokines by activated inflammatory cells including interleunkin-1beta (IL-1β), tumor necrosis factor alpha (TNF-α) and interferon gamma (IFNγ) [4]–[6]. It has been shown that exposure of beta-cells to these cytokines is sufficient to induce apoptosis [3], [4].

The c-Jun N-terminal Kinases (JNKs), also known as stress-activated protein kinases (SAPKs), are potently activated by pro-inflammatory cytokines and have been involved in cytokine-mediated beta-cell apoptosis [7]–[9]. Three JNK isoforms have been identified: JNK1, JNK2, and JNK3. JNK1 and JNK2 are ubiquitously expressed, while JNK3 was found to be restricted to the brain and testis [10], [11]; we however recently described high expression and functional role of this isoform in pancreatic islet cells [12]. Despite their high structural homology, the JNK isoforms have distinct biological functions. Genetic disruption of *Jnk1* is associated with insulin resistance and obesity [13], while *Jnk2* disruption partially protects Non-Obese Diabetic (NOD) mice from destructive insulitis [14]. While *Jnk3* knockout animals have not been studied for metabolic disorders, we provided evidence that JNK3 is protective against cytokine-induced apoptosis in an insulin-secreting cell line [12].

Several studies have shown that activation of JNK1 or JNK2 leads to inhibition of the pro-survival Akt (also called protein kinase B (PKB)) pathway and sensitizes pancreatic beta-cells to death [15]–[18]. Conversely, JNK blockade enhances Akt signaling and improves beta-cell survival [17]. It therefore seems that the JNK and Akt signaling pathways might cross-talk to determine the fate and function of the beta-cells in response to extracellular stimuli.

Three Akt (Akt1, Akt2, and Akt3) isoforms have been described, and they all share structural similarities; they however differ in their expression profiles and functions [19]–[21]. Akt1 is the major isoform ubiquitously expressed, while Akt2 is less abundant, except in insulin responsive tissues [22], [23]. The third isoform Akt3 has been described mostly in brain, testis and beta-cells [24]. Emerging evidence indicates that Akt controls beta-cell proliferation, survival, insulin synthesis and secretion [16], [25], [26]. *Akt1*-deficient mice have normal carbohydrate metabolism but show growth defects [20], [27]. Importantly, *Akt2*-deficient mice develop mild to severe diabetes with high beta-cell loss [28], [29]. It has been postulated that this high beta-cell loss results from an increased propensity of *Akt2*-null cells to die from apoptotic stimuli.

**Figure 1:**
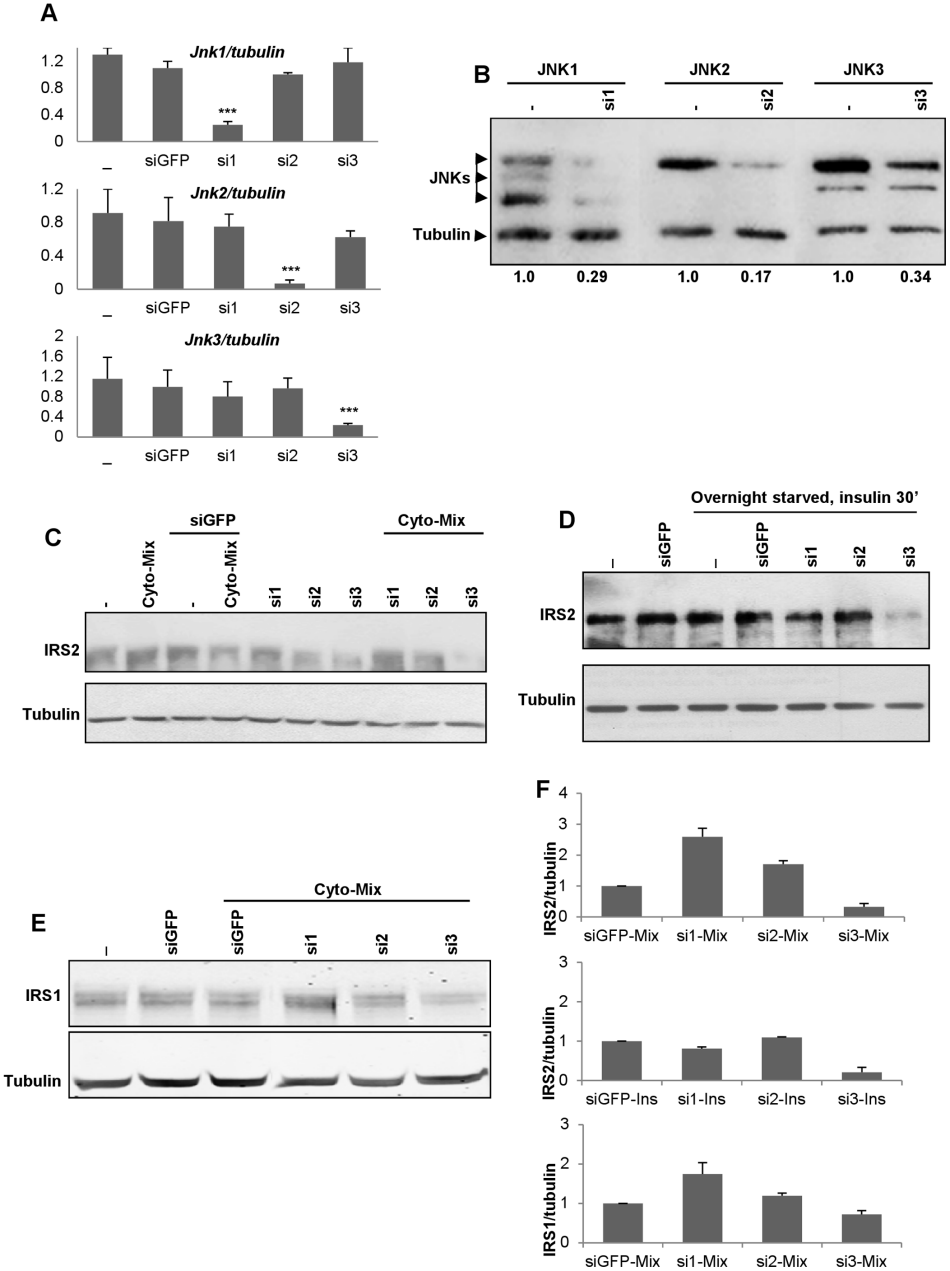
Efficiencies of the siRNAs. (**A**) INS-1E cells were transiently transfected with *Jnk1* (si1), *Jnk2* (si2), and *Jnk3* (si3) siRNAs for 2 days and total RNAs were prepared and used in quantitative real-time PCR experiments. GFP siRNA (siFGP) was used as a transfection control. Data were normalized to tubulin, and represent the means±SD of three independent experiments performed in triplicates. ***p<0.001 (**B**) JNK protein knockdowns were evaluated by western blotting using specific antibodies against JNK1, JNK2, and JNK3 as described previously (12). Densitometric values for the JNKs bands normalized to tubulin were shown below the blot; untreated cells (control) values were set to 1. Data are the means±SD of four independent experiments repeated in duplicates. ***p<0.001 (**C,D**) Cells transfected with *Jnk* (si1, si2, si3), or GFP (*siGFP*) siRNAs were exposed to (**C**) cytokines (4 hrs) or (**D**) overnight starved before being treated with insulin (30′). IRS2 protein expression levels were determined by western blot analysis. Equal protein loading was assessed by blotting membranes with an antibody against tubulin. (**E**) similar data as in (**C**), but showing IRS1 protein expression. (**F**) Graphical presentations summarizing the effects of the different *Jnk* siRNAs *vs* siGFP in stimulated conditions; control values are set to 1. Data are the means±SD of four independent experiments repeated in duplicates. **p<0.01 for cyto-Mix-siGFP *vs* cyto-Mix-si1 or cyto-Mix-si2 for (**C**), **p<0.01 for siGFP-Ins *vs* si3-Ins, and **p<0.01 for cyto-Mix-siGFP *vs* cyto-Mix-si1 and *p<0.05 for cyto-Mix-siGFP *vs* cyto-Mix-si2 (**E**).

A major regulator of Akt signaling in insulin-secreting cells is insulin itself that binds to the insulin receptor (IR) before recruiting the Insulin Receptor Substrates (IRSs) [30]–[32]. In turns, the IRSs mediate phosphoinositide3-kinase (PI3-K) activation and subsequent generation of phosphatidylinositol phosphate3 (PIP3) that binds and recruits Akt to the plasma membrane [33]. Full activation of Akt involves phosphorylation of both Threonine 308 (Thr^308^) and Serine 473 (Ser^473^) residues by different protein kinases [34], [35].

Akt activity is negatively regulated by two mechanisms: indirectly by dephosphorylation of the lipid PIP3 product by the protein phosphatase PTEN (phosphatase and tensin homolog) [36], [37], and by direct dephosphorylation of Akt by specific phosphatases, the Pleckstrin homology and leucine rich repeat protein phosphatases (PHLPPs) [38]. PTEN negatively regulates the intracellular levels of PIP3 in cells and functions as a tumor suppressor by regulating Akt signaling pathway [39]. The PHLPPs is a recently identified group of two protein phosphatases, PHLPP1 and PHLPP2 that inhibit several protein kinases including Akt. Both PHLPP1 and PHLPP2 have been shown to directly dephosphorylate and therefore inactivate Akt isoforms at one of two critical phosphorylation sites required for their activation [40]. PHLPP2 is able to dephosphorylate Akt1 at Ser^473^ whereas PHLPP1 preferentially dephosphorylates Akt2 [38], [40].

Akt acts through a wide array of downstream protein substrates involved in apoptosis, mitogenesis, cell proliferation and survival. Among the downstream targets of Akt are the Ser/Thr glycogen synthase kinase3β (GSK3β), and the members of the Forkhead BoxO (FoxO) family of transcription factors [41], [42]. Akt mediates cell survival through the phosphorylation of GSK3β which has been proposed as a promising target for beta-cell protection [43]. The phosphorylation of GSK3β by Akt (inhibits kinase activity) positively affects beta-cell mass and function while its dephosphorylation (kinase activation) enhances beta-cell death [44], [45]. The transcription factors FoxO consist of three members; FoxO1, FoxO3A, and FoxO4, which are all inactivated by Akt [46]. In pancreatic beta-cells, FoxO1 is predominantly expressed while FoxO3A is expressed at a lower level. Activation of Akt signaling mediates the phosphorylation of the FoxO factors which leads to the nuclear exclusion and then inhibition of the FoxO transcriptional program [47], [48]. In pancreatic beta-cells, it has been shown that FoxO3A specifically controls basal expression of IRS2; this participates to the maintenance of a normal beta-cell mass and function [49].

We have recently shown that JNK3, in contrast to JNK1 and JNK2, has a protective effect in pancreatic beta-cells [12]. We here propose that JNK3 mediates at least partly its protective effect against cytokines through functional preservation of the anti-apoptotic IRS2/Akt2 signaling pathway.

## Methods

### Cell Culture

The INS-1E cell line [50] was grown in RPMI-1640 medium (Invitrogen, Basel, Switzerland) containing 10% (vol/vol) heat-inactivated fetal bovine serum (FBS) supplemented with 1 mmol/l sodium pyruvate, 10 mmol/l HEPES (pH 7.6), and 50 μmol/l β-mercaptoethanol. Cells were incubated in a humidified atmosphere of 5% CO2 at 37°C.

### Cell Transfection and Treatments

Cells were incubated overnight at a density of 0.6×10^6^ in six-well plates with antibiotic free medium. Small interfering RNA (siRNA) duplexes targeting *Jnk1*, *Jnk2*, *Jnk3*, or the green fluorescent protein (GFP) were mixed with Lipofectamine^TM2000^ reagent according to the manufacturer’s instructions (Invitrogen, Basel, Switzerland). siRNA-Lipofectamine complexes were added to the cells and incubated for 2 days. Cells were then treated with a cocktail of cytokines: recombinant rat IL-1β (10 ng/ml, R&D systems, Minneapolis, MN, USA), TNF-α (10 ng/ml, Sigma-Aldrich, Switzerland), and IFNγ (100 ng/ml, Sigma-Aldrich, Switzerland) at the indicated times (see legend figures). For experiments aimed at characterizing insulin-signaling, cells were starved (serum-free) overnight in media supplemented with 2 mmol/l glucose. Cells were then stimulated with recombinant human insulin (100 nmol/l, Sigma-Aldrich, Switzerland) for 30 minutes and processed for protein extract preparations.

### Western Blotting

Cells were lysed in cold lysis buffer [51] and the protein extracts were recovered by centrifugation (at 14,000 rpm for 30 minutes) at 4°C. Equal quantities of total protein lysates were resolved by SDS-PAGE and electroblotted onto nitrocellulose membranes. The blots were probed overnight with primary antibodies against: JNK1, JNK2, JNK3, Akts, PTEN, IRS1, IRS2, FoxO3A, phospho-GSK3β, phospho-Akt1, phospho-Akt2; phospho-FoxO (1∶1,000; Cell Signaling Technology, MA, USA), PHLPP1 (1∶1,000 Millipore) and PHLPP2 (1∶200; Biotechnology, Santa Cruz, CA, USA). Equal protein loading was ascertained by blotting membranes against tubulin (1∶5,000; Sigma-Aldrich, Switzerland). Anti-rabbit or anti-mouse horseradish peroxidase-conjugated secondary antibodies were used to detect proteins with an enhanced chemiluminescence (ECL) reaction system (Pierce).

### RNA Preparation and Northern Blot Analysis

Total RNA was extracted using a commercial kit from Qiagen (RNeasy Mini-kit; QIAGEN AG, Basel, Switzerland). 1 µg of the prepared RNA was used for a single-strand cDNA synthesis by the “Transcriptor” high fidelity reverse transcriptase enzyme and performed according to the detailed instructions provided by the manufacturer (Transcriptor High Fidelity cDNA Synthesis Kit, Roche Diagnostics AG, Switzerland). *Jnk1, Jnk2, Jnk3,* and *tubulin* mRNA expressions were quantified using the standard LightCycler 480 SYBR Green I Master procedure according to the manufacturer’s instructions (LightCycler, 480 SYBR Green I Master, Roche Diagnostics AG, Switzerland). The sequences of the *Jnk1*, *Jnk2, Jnk3* or *tubulin* primers were previously described [12].

### Data Analysis

All experiments were performed a minimum of three times in duplicates (i.e. n = 3–5). Data are shown as means±SD. Statistical significances were calculated either by ANOVA or two-tailed *t* test for single comparisons.

## Results

### JNK3 Controls IRS2 Protein Content in Insulin-secreting Cells

IRS2 promotes beta-cell growth and survival and we have shown that cells with reduced JNK3 expression undergo spontaneous apoptosis [12]. We therefore wanted to determine whether JNK3 might control IRS2 in insulin-secreting cells. To this end, INS-1E cells were transfected with siRNAs targeting selectively each one of the three individual *Jnks* and RNA and protein extracts were prepared for RT-PCR and western blot analysis. *Jnk1* siRNA significantly reduced *Jnk1* (77% decrease) without interfering with *Jnk2* or *Jnk3* mRNA expression. Similarly, *Jnk2* (91.5% decrease), and *Jnk3* (76% decrease) siRNAs specifically decreased expression of their respective mRNAs (Fig.1A). The GFP siRNA used as a transfection control has no significant effect on the mRNA expressions on any of the three *Jnk* isoforms (Fig.1A). The different *Jnk* siRNAs were also tested at the protein level by western blot analysis using JNK isoform-specific antibodies (see our previous paper for a detailed analysis of the specificity of the antibodies used (12)). *Jnk1*, *Jnk2*, and *Jnk3* siRNAs reduced the protein expression of their respective JNK isoform by 71%, 83% and 66%, respectively (Fig.1B).

As shown in Figures 1C to D, JNK3 silencing markedly decreases IRS2 protein content, especially in presence of cytokines (Fig.1C), or following over-night starvation of cells (this stress results in activation of JNKs that reach similar levels to those obtained with the cytokines-mix used in this study, data not shown) (Fig.1D); in contrast, JNK1 silencing enhances IRS2 content (Fig.1C). We also observed a comparatively slighter decrease of IRS1 expression, but this did not reach statistical significance (Fig.1E). However, silencing JNK1 positively regulates IRS1 (Fig.1E/F).

### JNK3 Specifically Regulates Akt2 Phosphorylation in Insulin-secreting Cells

**Figure 2 pone-0035997-g002:**
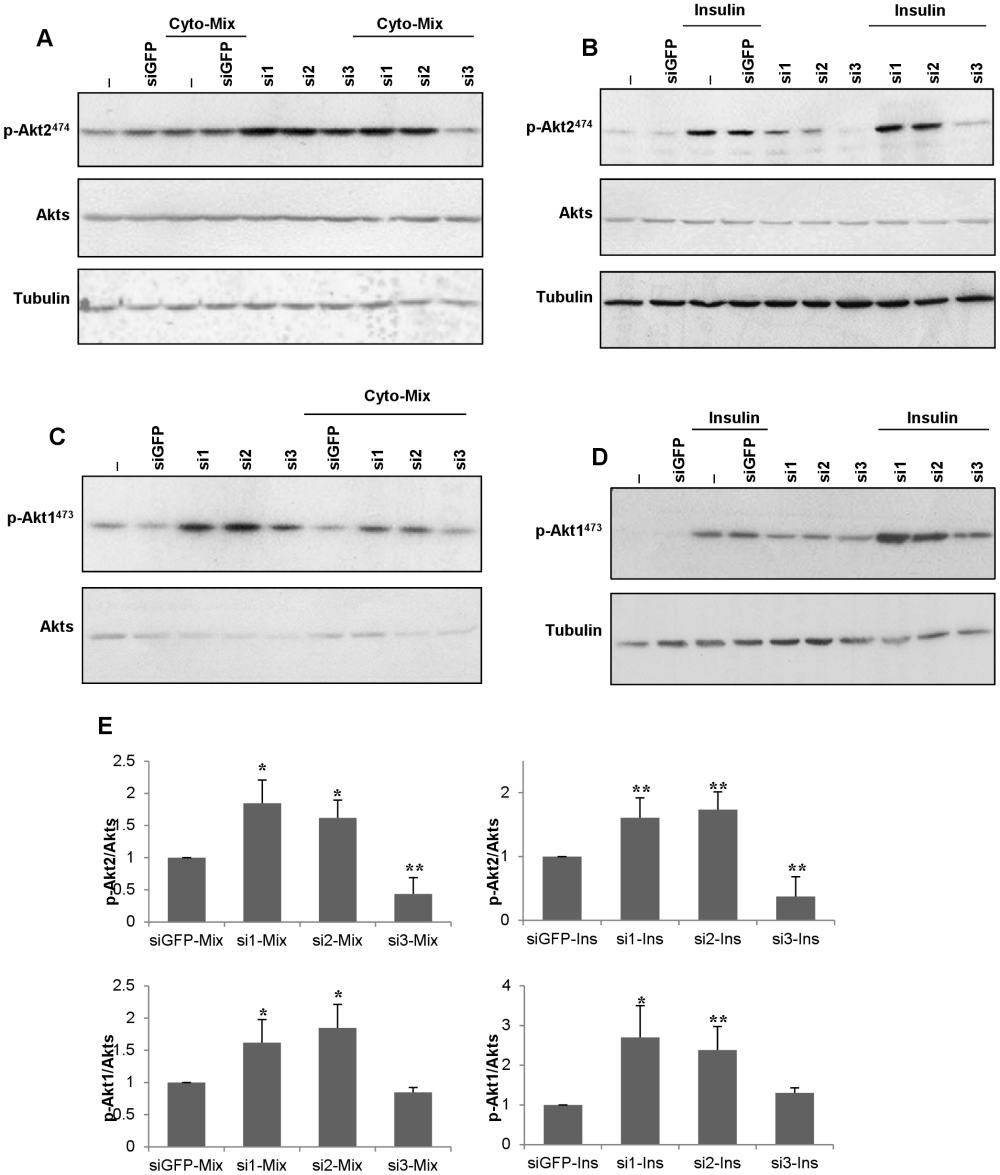
Effect of JNK silencing on Akts phosphorylation. Cells were transfected with *Jnks* (si1, si2, si3) or GFP (siGFP) siRNAs for 2 days. Transfected cells were (**A,C**) treated with cytokines (4 hrs) or (**B,D**) overnight starved then treated with insulin (30′). Protein extracts were used for western blotting with anti-phospho-Akt2 (p-Akt2^474^) and anti-Akts antibodies (**A,B**), or anti-phospho-Akt1 (p-Akt1^473^) and anti-Akts antibodies (**C, D**). Equal protein loading was assessed by blotting membranes with an antibody against tubulin. (**E**) Graphical presentations summarizing the effects of the different *Jnk* siRNAs *vs* siGFP in stimulated conditions; control values are further set to 1. The data are the means±SD of three independent experiments. Significant differences were obtained for p-Akt2/p-Akt1 in si1 and si2 *vs* p-Akt2/p-Akt1 (siGFP) in cytokines (*P<0.05) or starved-insulin treated cells (*P<0.05 or **p<0.01).

As for Akt1, the Akt2 isoform regulates beta-cell growth and survival; however, Akt2 distinctively controls glucose metabolism as *Akt2*-deficient mice develop diabetes [29]. Accordingly, we aimed to examine whether individual JNK silencing (specifically JNK3) could affect the activation profile of Akt2 or Akt1. As expected from the loss of IRS2 expression, decreased JNK3 efficiently inhibited insulin-induced Akt2 activation and caused disruption of insulin signaling (Fig.2). Western blot analysis indicates that JNK3 knockdown strongly inhibits Akt2^ser474^ phosphorylation (hence its activation) in cytokine-treated INS-1E cells (Fig.2A). Increase in Akt2 phosphorylation levels are evident following JNK1 or JNK2 knockdown in insulin or cytokine-treated cells (Fig.2A/B). In all tested condition, there are no changes in the protein expression levels of total Akts (Fig.2A/B).

### JNK1 or JNK2 Silencing Modulates the Phosphorylation of Akt1 in Insulin-secreting Cells

We next examined whether individual JNKs could distinctly interfere with Akt1 phosphorylation in response to insulin and cytokines stimuli. Our data show that JNK1 or JNK2 silencing enhanced Akt1^ser473^ phosphorylation at basal state or following cytokines (Fig.2C) or overnight starvation followed by insulin (Fig.2D) treatment. However, no major effect is observed in cells with reduced JNK3 (Fig.2C/D/E). The protein expression levels of total Akts remain unchanged in all condition tested (Fig.2).

### JNK Silencing Modulates the Activity of the Downstream Substrates of Insulin Signaling GSK3β and FoxO in Cytokine-treated Cells

**Figure 3:**
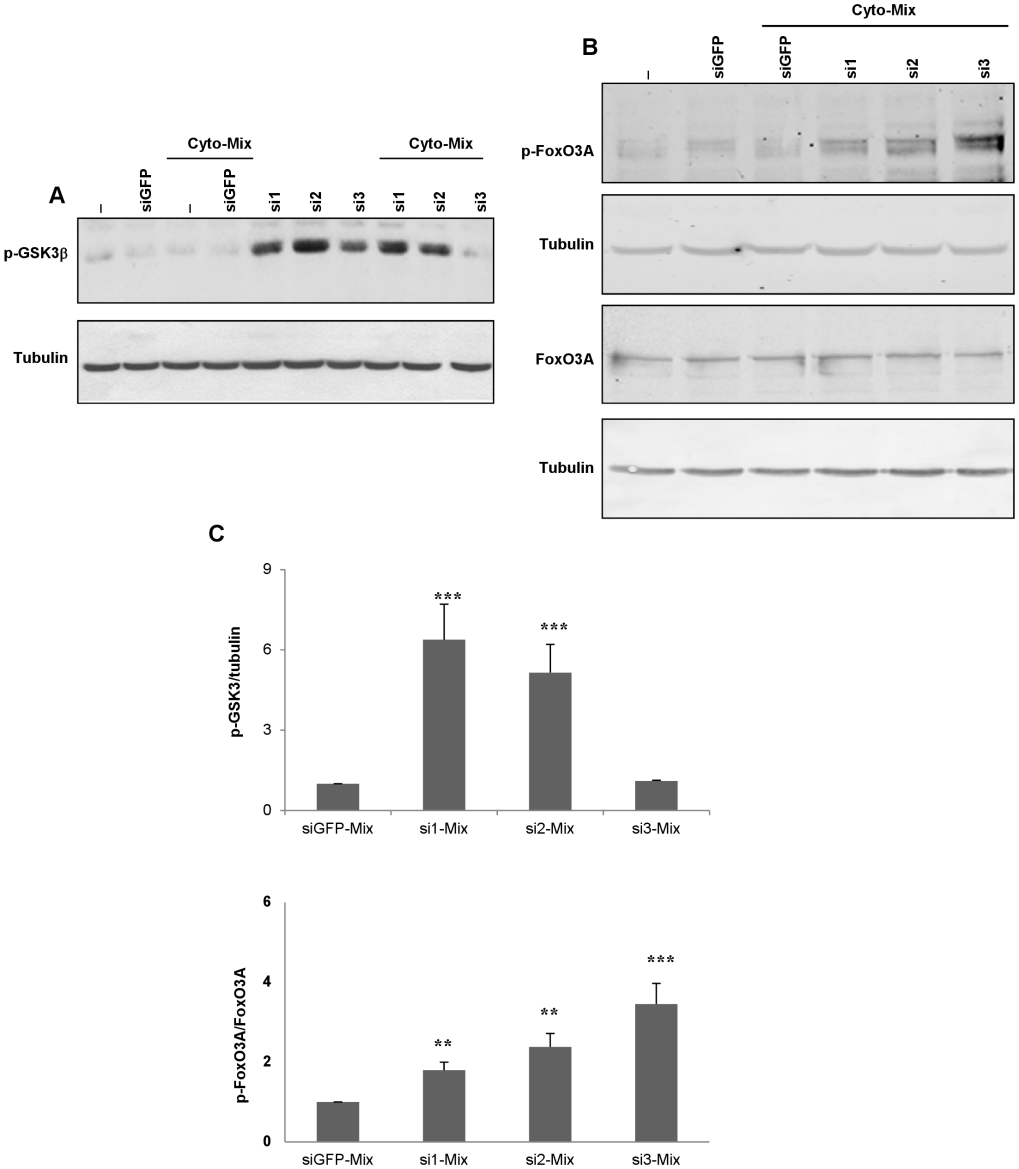
Effect of JNK silencing on downstream substrates of insulin signaling. Cells were transfected with *Jnks* (si1, si2, si3) or GFP (siGFP) siRNAs for 2 days and then exposed to cytokines (4 hrs). Western blot analysis was performed to determine (**A**) phospho-GSK3β or (**B**) phospho-FoxO3A and FoxO3A. Equal protein loading was assessed by blotting membranes with an antibody against tubulin. (**C**) Graphical presentations summarizing the effects of the different *Jnk* siRNAs *vs* siGFP in cytokine-treated cells; control values are set to 1. The data are the means±SD of three independent experiments. (**P<0.01) for cyto-Mix-siGFP *vs* cyto-Mix-si1, and cyto-Mix-si2. (***p<0.001) for cyto-Mix-siGFP *vs* cyto-Mix-si3. Significant differences were obtained for phospho-GSK3β in si1, si2, and si3 *vs* siGFP (***p<0.001).

It has been shown that Akt signaling acts on many pro-apoptotic targets in beta-cells, including the kinase GSK3β [44] and the transcription factors FoxO [46]. We first determined the activity of GSK3β following JNK silencing. Western blot analysis indicates that JNK1 or JNK2 silencing enhanced the phosphorylation of the GSK3β kinase (reduced activity) at both basal state and after cytokines treatment (Fig.3A). In contrast, JNK3 silencing has no effect on GSK3β activity compared to control conditions (Fig.3A/C).

In beta-cells, the PI3K-Akt pathway is a major upstream modulator of the activity of the transcription factors FoxO, which blocks their function by phosphorylation [46]. Recently, it has also been shown that FoxO3A controls IRS2 transcription in beta-cells [49]. To gain further insights into the regulatory mechanisms that may control the observed shutting-off of the IRS2 signaling pathway when JNK3 is silenced, we investigated the expression levels and phosphorylation status of the transcription factor FoxO3A after cytokine exposure. Western blot experiments show that JNK3 silencing is most efficient at enhancing the phosphorylation of FoxO3A (therefore inhibiting its activity) (Fig.3B/C).

### JNK3 does not Control the Activity of the Phosphatases PTEN and PHLPPs in Insulin-secreting Cells

**Figure 4:**
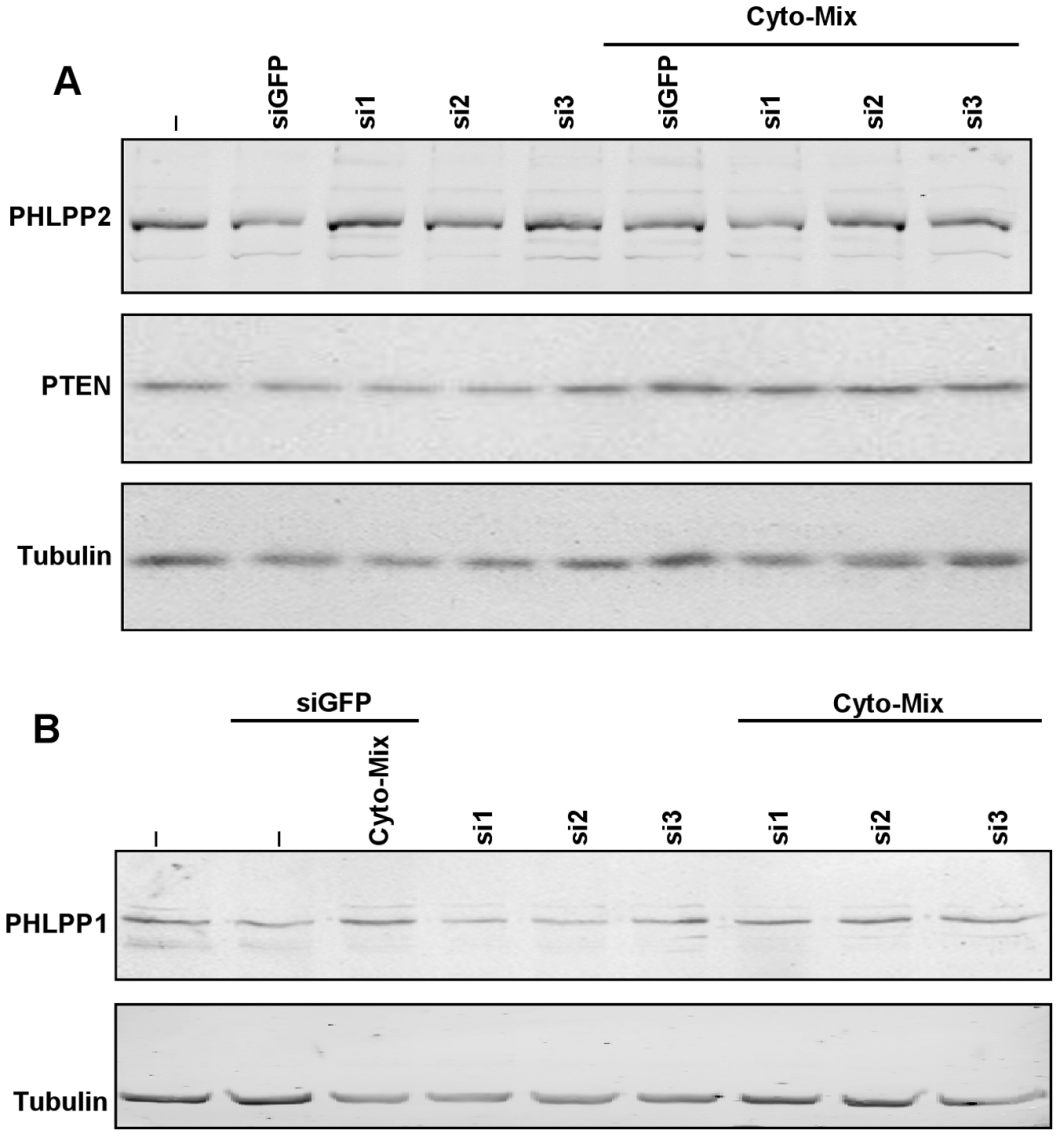
Expression of different regulators of the insulin-signaling pathway. Protein expression levels of the phosphatases (**A**) PTEN, PHLPP2, and (**B**) PHLPP1 were studied in conditions similar to those described in Figs.1 to [Fig pone-0035997-g002] 3. No relevant alterations of the protein expression levels in any condition could be demonstrated.

We also studied the expression levels of the phosphatases PTEN (Fig.4A) and PHLPP1/2 Fig.4A/B) that are known to regulate Akts activations. None of the parameters studied appeared to be influenced by JNK3 silencing in the INS-1E cell-line exposed to cytokines.

## Discussion

Pro-inflammatory cytokines have been shown to mediate beta-cell apoptosis through a mechanism that appears to involve the activation of the JNKs [8], [9]. Three JNK isoforms (JNK1, JNK2 and JNK3) have been described which are all expressed in insulin-secreting cells, and we have shown that in contrast to JNK1 and JNK2 whose activation is clearly pro-apoptotic, JNK3 has an unexpected role in preserving beta-cells against a number of different insults including cytokines [12]. We had postulated that these differential effects might be linked to the different sub-cellular localization of these isoforms: whereas JNK1 and JNK2 are mainly cytosolic, JNK3 is exclusively nuclear [12].

We here show that silencing of JNK3 leads to a marked reduction in IRS2 expression and signaling. The mechanism behind this regulation remains uncharacterized (but see below), but it implies that JNK3 is essential to preserve IRS2 expression, especially when cytokines are present (Fig.1). In contrast, silencing of JNK1 or JNK2 leads to an increased Akt signaling (Fig.2), an effect that is certainly linked to a decreased Ser/Thr phosphorylation of the IRS proteins by the lowered content of the cytosolic JNKs in these conditions [18], [52] (and hence an improved ability of the non-phosphorylated IRSs to bind to the IR). Conversely, JNK3, which is exclusively nuclear, is not expected to have direct access to the IRS proteins, and may only regulate them either at the transcriptional level or indirectly.

The role of IRS2 in beta-cell growth and survival has been well studied *in vitro* using primary pancreatic islets, and *in vivo*. Mice with full deletion of *Irs2* show peripheral insulin resistance and islet cell loss that progress to diabetes [53]. Moreover, mice with deletion of the *Irs2* gene specifically into pancreatic beta-cells develop glucose intolerance, and reduced beta-cell mass [54]. Therefore, IRS2 which appears to regulate peripheral response to insulin action also controls pancreatic beta-cell mass [55]. Conversely, signal transduction via IRS1 is less critical for beta-cell growth and survival as mice lacking *Irs1* did not become diabetic because of an adequate expansion of beta cell mass in presence of IRS2 [53]. In our conditions, reducing JNK3 did not show a significant decrease of IRS1 protein which indicates specific regulation of IRS2 by the JNK3 isoform. In contrast, we observed a slight increase in IRS1 expression levels when silencing JNK1.

As expected from the loss of IRS2 expression, our study shows that JNK3 suppression further inhibits Akt2 phosphorylation (phospho-Ser^474^) by insulin. Akt2 (which lays immediately downstream of IRS2 in the insulin signaling cascade) has been shown to control beta-cell growth and survival as well; it distinctively regulates glucose metabolism as *Akt2*-null mice develop severe diabetes with high loss of beta-cell mass, a phenotype clearly similar to the one observed with *Irs2*-null mice [28], [29], [53]. Hence, we may link the protective action of JNK3 in insulin secreting cells to its preservative role on the IRS2/Akt2 signaling pathway.

Akts may affect survival directly by regulating the activity (post-translational regulation) of their target substrates or indirectly by eliciting gene expression (transcriptional regulation). In beta-cells, GSK3β is a direct substrate of the PI3K-Akt pathway and its down regulation (increased phosphorylation) can protect cells from death [44], [56], [57].

Akts also modulate (by phosphorylation) the activity of the FoxO1 and FoxO3A transcription factors and block their translocation into the nucleus [46]. In beta-cells, the exact role of the FoxO proteins is not fully characterized [58]. It has been shown that decreasing levels of FoxO1 restore Pancreatic and Duodenal homeobox1 (PDX1) expression and nuclear localization and rescue the loss of beta-cells in *Irs2*-deficient mice, suggesting that FoxO1 and PDX1 can mediate proliferative signals induced by Akts [47], [59]. Alternatively FoxO3A but not FoxO1 has been shown to regulate *Irs2* expression [49]. In our conditions, JNKs silencing reduced FoxO3A activity (enhanced phosphorylation) particularly after JNK3 silencing. On the other hand, silencing JNK1 or JNK2 decreased FoxO1 activity, while its activity increased when JNK3 is silenced (data not shown). Activated FoxO1 may trigger the transcriptional machinery to induce the expression of relevant genes to inhibit beta-cell survival [47]. The decrease in FoxO3A activity while Akts activities are low (because of reduced JNK3 in presence of cytokines) may participate to the observed defect in IRS2 expression.

We have published previously that silencing of JNK3 aggravates the down-regulation of insulin mRNA levels caused by cytokines. Earlier studies have indicated that stress-mediated activation and stabilization of c-Jun suppresses insulin gene transcription by affecting the transactivation potential of the *E2A* gene products on the insulin promoter [60]. We have shown that JNK3, but not JNK1 or JNK2, silencing potently increases c-Jun levels, an effect that is in line with the specific nuclear localization of the kinase; it is therefore conceivable that the decrease in insulin mRNA expression observed in conditions of low JNK3 is mediated through an increase in c-Jun expression and/or stability [12]. In contrast, JNK1 and JNK2 with their mainly cytosolic localization do affect neither c-Jun levels nor insulin expression. With respect to IRS2 expression, it is known that both *Irs2* mRNA and protein are short-lived (with mRNA and protein half-lives of 90 min and 2 h, respectively), and thus IRS2 expression appears to be mainly regulated at the transcriptional level [61]; this is fully compatible with a transcriptional regulation of IRS2 by the nuclear JNK3 through regulation of FoxO3A. These data therefore reinforce our previous hypothesis, that stated that it might be the sub-cellular localizations of the different JNK isoforms that is predominant in governing the cellular response: JNK1 and JNK2 may lead to predominantly cytosolic responses (for example by binding to and phosphorylating and blocking the IRS proteins), while JNK3 will impact on nuclear responses (eg c-Jun expression or stability, transcriptional regulation of IRS2, etc). An important consequence of these recent works is that the JNK 1 and 2 probably mediate apoptosis mainly through cytosolic modifications of pre-existing proteins, while JNK3 appears to have a protective role which is essentially nuclear (transcriptionally) mediated. These conclusions might help understanding why previous attempts at characterizing the transcriptional effectors of apoptosis regulated by JNK were often disappointing.

In summary, we described here that expression of IRS2 is under the specific control of JNK3 in insulin-secreting cells. Hence, JNK3 appears to maintain the IRS2/Akt2 signaling module which is required to preserve beta-cell function and mass. Microarray studies using islet cells lacking *Jnk3* will establish the panel of genes that are regulated by JNK3 in pancreatic beta-cells (under investigation). Some of these genes might reveal new protective routes used by beta-cells to preserve their mass or function.
